# *Batrachochytrium salamandrivorans* not detected in U.S. survey of pet salamanders

**DOI:** 10.1038/s41598-017-13500-2

**Published:** 2017-10-13

**Authors:** Blake Klocke, Matthew Becker, James Lewis, Robert C. Fleischer, Carly R. Muletz-Wolz, Larry Rockwood, A. Alonso Aguirre, Brian Gratwicke

**Affiliations:** 1 Department of Environmental Science and Policy, George Mason University, Fairfax, Virginia 22030 United States of America; 2 Smithsonian Conservation Biology Institute, National Zoological Park, Washington, DC 20008 United States of America; 3 Center for Conservation Genomics, Smithsonian Conservation Biology Institute, National Zoological Park, Washington, DC 20008 United States of America; 4 Department of Biology and Chemistry, Liberty University, Lynchburg, Virginia 24515 United States of America; 5 Rainforest Trust, Warrenton, VA 20187 United States of America; 6 Department of Biology, George Mason University, Fairfax, Virginia 22030 United States of America

## Abstract

We engaged pet salamander owners in the United States to screen their animals for two amphibian chytrid fungal pathogens *Batrachochytrium dendrobatidis* (*Bd*) and *B*. *salamandrivorans* (*Bsal*). We provided pet owners with a sampling kit and instructional video to swab the skin of their animals. We received 639 salamander samples from 65 species by mail, and tested them for *Bd* and *Bsal* using qPCR. We detected *Bd* on 1.3% of salamanders (95% CI 0.0053–0.0267) and did not detect *Bsal* (95% CI 0.0000–0.0071). If *Bsal* is present in the U.S. population of pet salamanders, it occurs at a very low prevalence. The United States Fish and Wildlife Service listed 201 species of salamanders as “injurious wildlife” under the Lacey Act (18 U.S.C. § 42) on January 28, 2016, a precautionary action to prevent the introduction of *Bsal* to the U.S. through the importation of salamanders. This action reduced the number of salamanders imported to the U.S. from 2015 to 2016 by 98.4%. Our results indicate that continued precautions should be taken to prevent the introduction and establishment of *Bsal* in the U.S., which is a hotspot of salamander biodiversity.

## Introduction

Emerging fungal pathogens are an increasing threat to global biodiversity^1–3^. The pathogenic amphibian chytrid fungus *Batrachochytrium dendrobatidis* (*Bd*), is a major driver of global amphibian declines and extinctions^4–6^. From 2010–2013, catastrophic enigmatic declines of fire salamanders (*Salamandra salamandra*) in the Netherlands resulted in the description of a second species belonging to the genus *Batrachochytrium*, the salamander-eating fungus *B*. *salamandrivorans* (*Bsal*)^7^. Known host suitability and susceptibility to *Bsal* is restricted to salamanders (Urodela)^7,8^. Salamanders belonging to the family Salamandridae are highly susceptible to disease^7–9^. Although few frogs (Anura) have been tested in susceptibility trials, most have been found to be resistant to infection^7,8^. However, the common midwife toad (*Alytes obstetricans*) has been shown to maintain subclinical infections of *Bsal* for several weeks and may act as infection reservoirs^10^. It is hypothesized that *Bsal* is endemic to salamanders in Asia and was introduced to Europe via the pet trade^8^. This hypothesis is supported by recent *Bsal* detection at endemic levels in newt populations of Vietnam^9^. *Bsal* has now been detected in the environment in the Netherlands, Belgium, Germany, and Vietnam^9,11,12^, and in captive individuals in the United Kingdom and Germany^11,13^.

Salamanders from Asia represent the majority of salamander species traded internationally for the pet trade and pose a significant threat for the spread of disease^14–16^. The U.S. is a global salamander biodiversity hotspot and the threat of *Bsal* introduction via the pet trade generated significant concern^15,16^. As a precautionary approach to avoid *Bsal* introduction, on 28 January 2016, the U.S. Fish and Wildlife Service included 20 genera of salamanders representing 201 species in the “injurious wildlife” list under the Lacey Act (18 U.S.C. § 42). This was intended to restrict interstate transport and importation of injurious species (https://www.fws.gov/injuriouswildlife/salamanders.html), but recent legal determinations on the scope of the Lacey Act (18 U.S.C. § 42) authority for injurious wildlife may reduce these restrictions^17^. An urgent priority is to determine whether, in fact *Bsal* already exists in captive or wild salamanders in the U.S. The *Bsal* Task Force and Partners in Amphibian and Reptile Conservation’s Disease Task Team call for determining the prevalence and biosecurity risk of *Bsal* in captive collections^16^. Therefore, we initiated a citizen science project engaging pet salamander owners to screen their salamanders for *Bd* and *Bsal* to evaluate the prevalence of these pathogens in U.S. private salamander collections.

## Results

A total of 639 of 4,462 (13.8%) swabs were returned by mail. These 639 swabs were submitted by 56 private salamander owners from 29 states in the U.S. Returned swabs represented 65 species, 22 genera, and three families of salamanders (Ambystomatidae, Plethodontidae, and Salamandridae) (Table 1). Twelve of the genera, containing 40 species tested, are now prohibited from importation to the U.S. under the “injurious wildlife” provision of the Lacey Act (18 U.S.C. § 42). Of the 639 salamanders tested, 486 individuals (76.1%) belong to genera containing at least one species known to be susceptible to *Bsal* ^8,9^. Salamander species tested represent 73% of the species of salamanders imported to the U.S. from 2010–2016. Our return rate may have been low due to (i) the limited time window for returning the testing kits given the 12 January 2016 announcement listing 201 species of salamanders under the “injurious wildlife” provision and (ii) the return shipping costs not being covered.

**Table 1:** Summary of salamander species tested for *Bsal* and *Bd* in U.S. captive collections.

Family	Genus	Species	N	*Bsal*	*Bd*	Number Imported 2010–2016
Ambystomatidae	*Ambystoma*	*spp*.	109	0	1	1,762
Plethodontidae	*Aneides*	*aeneus*	4	0	0	—
	*Desmognathus*	*spp*.	6	0	0	—
	*Ensatina*	*eschscholtzii*	7	0	0	3
	*Eurycea*	*spp*.	4	0	0	—
	*Gyrinophilus*	*porphyriticus*	1	0	0	—
	*Hemidactylium*	*scutatum*	2	0	0	—
	***Plethodon***	*spp*.	16	0	0	—
	*Pseudotriton*	*ruber*	2	0	0	—
	*Urspelerpes*	*brucei*	1	0	0	—
Salamandridae	***Cynops***	*chenggongensis*	3	0	0	—
		*cyanurus*	42	0	0	3,493
		*ensicauda*	16	0	0	14
		*orientalis*	49	0	1	306,176
		*pyrrhogaster*	30	0	0	69,012
	***Ichthyosaura***	*alpestris*	36	0	0	—
	*Laotriton*	*laoensis*	1	0	0	30
	***Lissotriton***	*vulgaris*	7	0	0	—
	***Neurergus***	*crocatus*	15	0	0	325
		*kaiseri*	28	0	1	90
		*strauchii*	8	0	0	—
	***Notophthalmus***	*viridescens*	17	0	1	469
	***Paramesotriton***	*labiatus*	4	0	0	12,162
		*caudopunctatus*	1	0	0	—
		*chinensis*	6	0	0	1,301
		*deloustali*	1	0	0	8
		*hongkongensis*	14	0	0	190,011
	***Pleurodeles***	*nebulosus*	2	0	0	—
		*waltl*	25	0	2	207
	***Salamandra***	*salamandra*	40	0	0	8,509
	***Taricha***	*granulosa*	10	0	0	—
		*rivularis*	7	0	0	—
		*sierrae*	1	0	0	—
		*torosa*	15	0	0	—
	***Triturus***	*carnifex*	1	0	0	123
		*dobrogicus*	29	0	1	—
		*ivanbrueschi*	4	0	0	—
		*karelinii*	15	0	0	—
		*macadonicus*	3	0	0	—
		*marmoratus*	24	0	0	16
		*pygmaeus*	1	0	0	—
	***Tylototriton***	*kweichowensis*	11	0	0	1,704
		*verrucosus*	8	0	0	406
		*yangi*	13	0	0	—
**Total**	**22 genera**	**65 species**	**639**	**0**	**7**	**595**,**821**

Genera in bold text include species susceptible to *Bsal* and are listed as of January 28, 2016 in the “injurious wildlife” provision under the Lacey Act (18 U.S.C. § 42). A complete table with all species tested can be found in Supplementary Table S1.

We detected *Bd* on 1.3% of salamanders (95% CI 0.0053–0.0267) and did not detect *Bsal* (95% CI 0.0000–0.0071). Seven samples tested positive for *Bd*, but with low (<100) genomic equivalents that are likely associated with sub-clinical infections (Table 2). We are 95% confident that the maximum prevalence of *Bsal* in the U.S. captive salamander population is less than 0.71%, if it is in the U.S., and that the prevalence of *Bd* is between 0.53–2.67%.

**Table 2:** Clopper-Pearson 95% CI for prevalence of *Bsal* and *Bd* in captive pet collections.

Chytrid Fungus	Validated Number of Samples Tested	Number Positive	Proportion Infected	Clopper-Pearson 95% Confidence Interval	Probability of detecting at least one positive individual (assuming prevalence = 0.01)
*Batrachochytrium salamandrivorans*	537	0	0.0000	0.0000–0.0071	0.9955
*Batrachochytrium dendrobatidis*	537	7	0.0130	0.0053–0.0267	0.9955

This suggests that both *Bsal*, if present, and *Bd* are at low prevalence in pet collections in the U.S. Probability of detecting at least one positive individual assuming prevalence was *p* = 0.01 was calculated with the following equation D = 1-(1-*p*)^*n*^ after incorporating our confidence in DNA quality (see Methods).

## Discussion

*Bsal* has not been detected to date in captive collections or in wild populations in the U.S.^8,18,19^. Our results suggest that if *Bsal* is present in private captive U.S. salamander collections, it occurs at extremely low prevalence. The non-detection of *Bsal* in captive and wild U.S. salamanders suggests that *Bsal* has not yet been introduced into the U.S., justifying continued monitoring and precautions to prevent its spread. The prevalence of *Bd* was similar to other surveys of captive collections^20^.

We screened a diversity of native and non-native salamander species, with higher relative numbers of non-native species. Many of the non-native species we tested have not been imported in recent years indicating that hobbyists may be sustaining captive-bred populations of these animals in the U.S. Many species native to the U.S. were represented by low sample sizes, which may be due to laws protecting the collection of these species, complex captive husbandry and rearing, or low desirability in keeping native species as opposed to exotic species. *Cynops ssp*. and *Paramesotriton ssp*. represent 91.6% of individuals imported from 2010–2016 (Table 1), but compose 26.0% of salamanders tested. These species were often sold by wholesalers and chain pet stores, but may not be as popular in the community of hobbyists that responded to our swabbing request. Our sampling may have been biased towards more actively engaged hobbyists. The number of salamanders in private collections in the U.S. remains unknown.

On April 7, 2017, the United States Court of Appeals ruled that the “injurious wildlife” provision of the Lacey Act (18 U.S.C. § 42) does not restrict interstate transport of listed species in the continental U.S.^17^, which has implications for the potential introduction or spread of *Bsal* in the U.S. At the time of this publication, neither the United States Fish and Wildlife Service nor Department of Interior have issued official guidance on this court ruling, it is currently unclear if permit requirements or enforcement exists when transporting species listed as “injurious wildlife” across state borders. Interstate trade moratorium of the 201 salamander species listed as “injurious wildlife” on January 28, 2016 may no longer be in effect in the continental U.S. If this interpretation is upheld by the agencies, the ruling will allow for any animals that may be infected with *Bsal* or *Bd* to be transported across the U.S. state boundaries. This interpretation may also affect the interstate transport of other invasive taxa that are currently listed as “injurious wildlife”, i.e., Asian carp (*Hypophthalmichthys ssp*.) and Snakeheads (Channidae). In absence of the Lacey Act (18 U.S.C. § 42) regulating interstate transport of “injurious wildlife” species, enforcement is reliant upon state laws to prohibit the transport or introduction of invasive species. Invasive species are a significant threat to biodiversity, and this ruling sets a precarious precedent for the future of federal management of invasive species in the U.S. The Lacey Act of 1900 (18 U.S.C. § 42) was the first federal law protecting wildlife and was not written to protect wildlife from pathogens. Wildlife agencies with a mandate to manage and mitigate risks to wildlife involving emerging diseases would be better served by comprehensive wildlife health legislation.

Current import restrictions are limited to 201 species of salamanders known to be susceptible to *Bsal*. The listing of these species as “injurious wildlife” effectively halted importation of salamanders to the U.S. In 2015, there were 186 importations of salamanders containing 68,102 individuals. The declared value of all salamander imports in 2015 was $43,501 (USD). A total of 7 importations of salamanders containing 1,062 individuals occurred in 2016. Four of these importations contained species to be listed as “injurious wildlife” and were imported before the “injurious wildlife” listing went into effect on January 28, 2016. A total of 22 salamanders were imported after the “injurious wildlife” listing came into effect in 2016, one shipment contained 18 *Paramesotriton honkongensis*, an “injurious wildlife” species. Although the “injurious wildlife” listing of 201 salamander species reduced the volume of salamanders imported to the U.S., we do not have a comprehensive understanding of the potential for all urodelans, anurans or gymnophionans to act as *Bsal* hosts. One of the current limitations to verifying that an animal is pathogen free is that the diagnostic assays for *Bd* and *Bsal* use real-time qPCR which is (i) time intensive, (ii) requires specialized equipment, and (iii) requires trained personnel. The use of qPCR for diagnosing these pathogens makes it challenging to screen animals at ports of entry or sale. The recent development of lateral flow assay (LFA), a portable, rapid, inexpensive test to detect *Bd* and *Bsal* has potential to be an invaluable tool for detecting these pathogens^21^. This technology could be deployed for rapid testing of imported amphibians at port of entry, in private collections, zoos and in the field.

Communication and engagement with the amphibian hobbyist community is imperative to reduce the risk of pathogen introduction into the environment. Newts belonging to the genus *Cynops* are potential reservoirs for *Bsal* ^8^. Release of unwanted pets into the environment has led to the introduction of *C*. *pyrrhogaster* into the wild in Massachusetts^22^, and *C*. *orientalis* in Florida^23^, and presents a potential pathway for *Bsal* introduction into native salamander populations in the U.S. There are no proven mitigation methods for preventing the spread of *Bsal* once introduced into the environment, and *Bsal* introduction in the U.S. would likely be catastrophic for salamander biodiversity. Release of unwanted pets into the environment should never be considered an option. We encourage hobbyists to test their animals for *Bsal* and *Bd*, and disinfect wastewater and materials from enclosures before disposal to prevent pathogen introduction to the environment.

Our citizen science project introduced pet owners to the risks of these emerging pathogens and to biosecurity protocols that can reduce the risk of pathogen introduction to the environment. A *Bsal* Task Force (salamanderfungus.org) has been established to provide informational resources to respond to possible cases of *Bsal* in the U.S. Continued public outreach, salamander disease testing, preventing release of unwanted pets, and education about biosecurity should be a priority to prevent *Bsal* introduction.

## Methods

### Salamander Testing

Free testing for *Batrachochytrium salamandrivorans* and *Batrachochytrium dendrobatidis* was offered to salamander hobbyists through the Amphibian Survival Alliance website (http://www.amphibians.org/salamanderheros/) between November 2014 and January 2016. Experimental methods were approved by the Smithsonian National Zoological Park Institutional Animal Care and Use Committee approval #14–36. All methods were carried out in accordance to these guidelines and regulations. Testing kits were mailed to salamander hobbyists and included one of the following for each salamander: MW113 Swab (Medical Wire, United Kingdom), a pair of nitrile powder-free gloves, 1.5 ml microcentrifuge tube, and a plastic ziplock bag. A swabbing protocol, instructional video (https://www.youtube.com/watch?v=U5h5srxXAaY&), and data sheet were provided. Each salamander was captured in a clean plastic bag and swabbed 10 times on the ventral surface, five times on each foot, and five unilateral strokes on the tail. The swab was air-dried for 5 minutes, then placed in the 1.5 ml centrifuge tube and returned via mail with a corresponding data sheet noting the species and swab number. Participants were instructed to return their swabs before the 28 January 2016 interstate movement moratorium came into effect. After testing, participants were informed whether *Bsal* and *Bd* was detected on their salamanders. Hobbyists with salamanders that tested positive for *Bd* were advised to consult a veterinarian for treatment.

### DNA Extraction and qPCR

Dried swabs returned by mail were stored at −20 °C until extracted using a Qiagen DNeasy Blood and Tissue Kit (Germantown, MD, USA) with the manufacturer’s protocol. Real-time PCR procedures followed Blooi *et al*.^24^ using the CFX96 real-time system (Bio-Rad Laboratories, Hercules, CA), except that we assayed *B*. *dendrobatidis* and *B*. *salamandrivorans* independently and in duplicate samples per individual, rather than using the duplex approach, and a FAM-labeled STerC probe was used in place of a Cy5-labeled STerC probe. If only one of the duplicate samples tested positive (n = 639), the real-time PCR was repeated in duplicate to verify the *Bsal* or *Bd* status.

We verified that our protocol successfully extracted and amplified DNA, in the potential absence of amplification of *Bd* or *Bsal* by attempting to amplify a small section of salamander mitochondrial DNA. We designed the primers Salcytb1 (forward: 5′CAATGGCCCACACTAYACGA3′ and reverse: 5′TGCTGAGTGTGTGTCTGCTG3′) and Salcytb2 (forward: 5′AGGGGCCACAGTCATYACHA3′ and reverse: 5′CCTGTTGGGTTGTTTGABCC3′) to amplify an approximately 200 bp DNA fragment in the mitochondrial cytochrome b (ctyb) gene by using available salamander ctyb sequences on Genbank. We tested 95 individuals that represented multiple DNA extraction plates, 27 salamander species and 17 pet owners. Each 25-ul PCR assay consisted of 1.25U of AmpliTaq Gold DNA Polymerase (ThermoFisher), 2.5 uM MgCl_2_, 1x PCR Buffer, 200 nM dNTPs, 600 nM reverse primer, 600 nM forward primer, and 5 ul DNA template. PCR conditions were 95 °C for 10 m, followed by 45 cycles of 95 °C for 15 s, 51 °C for 30 s, 72 °C for 30 s, and a final extension (72 °C for 5 m).

### Data Analysis

We obtained Law Enforcement Management Information System (LEMIS) records of live salamander trade imports from 2015–2016 using a Freedom of Information Act request to the U.S. Fish and Wildlife Service to determine the effect of the “injurious wildlife” listing of 201 salamander species on the number of salamanders imported to the U.S (see Supplementary Table S2). Previously obtained and published LEMIS records of salamander importations from 2004–2014 were used to compare the relative proportion of species imported vs. maintained in captivity^16^.

We calculated Clopper-Pearson 95% Confidence Intervals for *Bd* and *Bsal* prevalence. We successfully amplified salamander ctyb DNA in 80/95 swabs, indicating that 84% of samples had good DNA quality. Samples that did not amplify had significantly lower DNA concentrations than those that amplified (Wilcoxon rank sum test, W = 54, p = 0.026). We did not observe a pattern in samples that did not amplify; other samples from the same pet owners amplified, and samples from the same salamander species sampled by other pet owners amplified. It is possible that instructions for swabbing salamanders were not properly followed for a small subset of individual salamanders, DNA degraded during shipping, or that substrate on the swab inhibited DNA extraction. We calculated our confidence intervals to incorporate our confidence in DNA quality (639 samples tested × 84% = 537 samples; Table 2). With no positive samples of *Bsal*, we assumed the prevalence was 1% for calculating the probability of detecting at least one positive individual^8^.

## Electronic supplementary material

Supplementary Material

Supplementary Table S2
