# Supplementary material for: Batrachochytrium salamandrivorans not detected in U.S. survey of pet salamanders
Source: Sci Rep. 2017 Oct 13;7:13132. doi: 10.1038/s41598-017-13500-2 (PMC5640657; doi:10.1038/s41598-017-13500-2)
Supplement: Supplementary file 1 — Supplementary Material [file 41598_2017_13500_MOESM1_ESM.pdf]

Supplementary Material for

Batrachochytrium salamandrivorans not detected in U.S. survey of pet salamanders

Blake Klocke*, Matthew Becker, James Lewis, Robert C. Fleischer, Carly R. Muletz-Wolz, Larry Rockwood, A. Alonso Aguirre, Brian Gratwicke

*Corresponding author (blake.klocke@gmail.com)

16 September 2017

Family	Genus	Species	N	*Bsal*	*Bd*	Number Imported 2010-2016
Ambystomatidae	*Ambystoma*	*gracile*	2	0	0	-
		jeffersonianum	2	0	0	-
		laterale	1	0	0	-
		maculatum	7	0	1	317
		mavortium	8	0	0	-
		mexicanum	18	0	0	1,392
		opacum	5	0	0	3
		talpoideum	1	0	0	-
		tigrinum	65	0	0	50
Plethodontidae	*Aneides*	*aeneus*	1	0	0	-
		flavipunctatus	1	0	0	-
		lugubris	2	0	0	-
	Desmognathus	*aeneus*	2	0	0	-
		folkertsi	1	0	0	-
		ocoee	2	0	0	-
		wrighti	1	0	0	-
	Ensatina	*eschscholtzii*	7	0	0	3
	Eurycea	*lucifuga*	1	0	0	-
		wilderae	1	0	0	-
		cirrigera	2	0	0	-
	Gyrinophilus	*porphyriticus*	1	0	0	-
	Hemidactylium	*scutatum*	2	0	0	-
	Plethodon	*cinereus*	3	0	0	-
		dunni	1	0	0	-
		glutinosus	5	0	0	-
		shermani	1	0	0	-
		wehrlei	3	0	0	-
		yonahlossee	1	0	0	-
		serratus	2	0	0	-
	Pseudotriton	*ruber*	2	0	0	-
	Urspelerpes	*brucei*	1	0	0	-
Salamandridae	***Cynops***	*chenggongensis*	3	0	0	-
		cyanurus	42	0	0	3,493
		ensicauda	16	0	0	14
		orientalis	49	0	1	306,176
		pyrrhogaster	30	0	0	69,012
	Ichthyosaura	*alpestris*	36	0	0	-
	Laotriton	*laoensis*	1	0	0	30
	Lissotriton	*vulgaris*	7	0	0	-
	Neurergus	*crocatus*	15	0	0	325
		kaiseri	28	0	1	90
		strauchii	8	0	0	-
	Notophthalmus	*viridescens*	17	0	1	469
	Paramesotriton	*labiatus*	4	0	0	12,162
		caudopunctatus	1	0	0	-
		chinensis	6	0	0	1,301
		deloustali	1	0	0	8
		hongkongensis	14	0	0	190,011
	Pleurodeles	*nebulosus*	2	0	0	-
		waltl	25	0	2	207
	Salamandra	*salamandra*	40	0	0	8,509
	Taricha	*granulosa*	10	0	0	-
		rivularis	7	0	0	-
		sierrae	1	0	0	-
		torosa	15	0	0	-
	Triturus	*carnifex*	1	0	0	123
		dobrogicus	29	0	1	-
		ivanbrueschi	4	0	0	-
		karelinii	15	0	0	-
		macadonicus	3	0	0	-
		marmoratus	24	0	0	16
		pygmaeus	1	0	0	-
	Tylototriton	*kweichowensis*	11	0	0	1,704
		verrucosus	8	0	0	406
		yangi	13	0	0	-
**Total**	**22 genera**	**65 species**	**639**	**0**	**7**	**595,821**

Supplementary Table S1: All salamander species tested for *Bsal* and *Bd* in U.S. Captive collections. Bolded genera contain species susceptible to *Bsal* and are listed as "Injurious" under the Lacey Act (18 U.S.C. 42).

Supplementary Information: Instructions for swabbing pet salamanders to test for *Batrachochytrium dendrobatidis* and *B. salamandrivorans*

How to swab a salamander for *Batrachochytrium salamandrivorans*

View an instructional video on how to swab a salamander here:
https://www.youtube.com/watch?v=U5h5srxXAaY&feature=youtu.be

Swabs that have been used on listed species under the new ruling must be sent earlier enough to arrive before January 28.

Please check the email that you registered with to obtain the data sheet or email bklocke@masonlive.gmu.edu

1) Make sure you have one MW113 swab and empty 1.5ml tube for each animal you plan to swab.
2) Pick up the individual salamander from its tank using clean powder-free nitrile disposable gloves, or an inverted new ziplock bag.
3) Place the salamander in a clean ziplock bag and gently hold it by its torso, not by its tail. We recommend swabbing the salamander immediately and leaving it in the ziplock bag for no longer than 30 minutes.
4) Open the sterile swab packet and remove the swab taking care that the tip does not touch anything other than the salamander or the inside of the ziplock bag. Gently run the swab 10 times back and forth along the belly, five times on each foot, and five unidirectional strokes on the tail starting from the cloaca and gently sliding it towards the tail tip.
5) Snap the tip off the swab directly into the 1.5ml tube, allow swab to air dry for 15 minutes once in tube, and then seal it.
6) Using a permanent marker label the tube with your name, the date, salamander species and animal number (if you are providing more than one swab).
7) Mail the tube and data sheet back to:

   Brian Gratwicke

   Center for Species Survival

   Smithsonian Conservation Biology Institute

   MRC 5503 PO Box 37012

   Washington DC 20013-7012

Please write down all the information on each tube on a separate sheet of paper and if possible, provide any supplementary information such as where you got the salamander, how long you have had it as a pet, or any other observations about its health.

This project is being conducted by Blake Klocke a student at George Mason University and swabs will be analyzed at the Smithsonian Conservation Biology Institute which is an Amphibian Survival Alliance Partner. bklocke@masonlive.gmu.edu

Supplementary Information: Internet link with basic biosecurity information and registration to receive testing kits for *Bd* and *Bsal*

Amphibian Survival Alliance: Be a Salamander Hero

http://www.amphibians.org/salamanderheros/
